# Effects of Wheat Germ Extract on the Severity and Systemic Symptoms of Primary Dysmenorrhea: A Randomized Controlled Clinical Trial

**DOI:** 10.5812/ircmj.19503

**Published:** 2014-08-05

**Authors:** Maryam Atallahi, Sedigheh Amir Ali Akbari, Faraz Mojab, Hamid Alavi Majd

**Affiliations:** 1 Department of Midwifery, Shahid Beheshti University of Medical Sciences, International Branch, Tehran, IR Iran; 2 Faculty of Nursing and Midwifery, Hamadan University of Medical Sciences, Hamadan, IR Iran; 3 Department of Midwifery, Faculty of Nursing and Midwifery, Shahid Beheshti University of Medical Sciences, Tehran, IR Iran; 4 Department of Pharmacognosy, Shahid Beheshti University of Medical Sciences, Tehran, IR Iran; 5 Department of Biostatistics, Faculty of Paramedicine, Shahid Beheshti University of Medical Sciences, Tehran, IR Iran

**Keywords:** Dysmenorrhea, Herbal Medicines, Wheat Germ

## Abstract

**Background:**

Primary dysmenorrhea is one of the most common disorders in women and the main cause of absenteeism from work and school.

**Objectives:**

Considering the anti-inflammatory properties of wheat germ, the present study examined the effects of wheat germ extract on the severity and systemic symptoms of primary dysmenorrhea.

**Patients and Methods:**

This triple-blinded clinical trial was performed on 80 employed women in hospitals affiliated with Hamadan University of Medical Sciences (Hamadan, IR Iran). Subjects were allocated to two groups of 45 patients. Three 400-mg capsules of wheat germ extract or placebo were used daily٫ between the 16th day of the menstrual cycle to the fifth day of the next menstrual cycle for two consecutive months. Pain intensity was measured by a visual analogue scale thrice a day and a four-point verbal rating scale was employed to assess systemic symptoms.

**Results:**

Pain severity decreased only in the wheat germ extract group (P < 0.001) and there was no statistically significant change in the placebo group. In the wheat germ extract group, the pain severity decreased from 4.701 at baseline to 1.120 at the second cycle. The reduction in total scores of systemic symptoms of dysmenorrhea was statistically significant only in the wheat germ extract group (P < 0.001) and there was not a statistically significant change in the placebo group. It revealed statistically significant differences in systemic symptoms associated with dysmenorrhea including fatigue, headache, and mood swings in experimental group.

**Conclusions:**

Wheat germ extract seems to be an effective treatment for dysmenorrhea and its systemic symptoms, probably because of its anti-inflammatory effects.

## 1. Background

Dysmenorrhea or painful menses is a common gynecological condition (1-3). It is commonly categorized into two types of primary and secondary dysmenorrhea based on pathophysiology. Primary dysmenorrhea is painful menses without organic diseases that refers to monthly suprapubic pain beginning between several hours before and a few hours after the onset of menstrual bleeding, and secondary dysmenorrhea is painful menses in association with an identifiable disease (4-6). Primary dysmenorrhea is associated with some symptoms such as nausea, vomiting, diarrhea, headache, fatigue, dizziness, and syncope (7, 8). Pain intensity in dysmenorrhea can be mild (pain that does not disturb daily activities and does not require painkillers), moderate (pain that slightly interferes with daily routines but can be managed with painkillers), and severe (pain that completely prevents daily life activities) (9).

According to previous studies, 70% to 91% of young girls have dysmenorrhea (10) and about 14% to 23% experience severe symptoms. Dysmenorrhea is the leading cause of absenteeism from the work or school and reduced quality of life (7, 10-13).

The most important cause of primary dysmenorrhea is increased production of prostaglandins (14-17). Various methods including local heat therapy, herbal therapy, reflexology, acupuncture, and the administration of thiamine, vitamin E, magnesium supplementation, acetaminophen, aspirin, and oral contraceptives have been suggested to control and treat dysmenorrhea (3, 18-25). Herbal therapy is a type of traditional medicine (2) that uses herbal medicines as more reliable, more effective, and more economical alternatives for chemical medications (26). Naturally occurring agents used to treat dysmenorrhea include *Rosa damascena* (27, 28), fenugreek seed (2), fennel (29, 30), Shirazi Thymus (4), and valerian (31). Among herbal medicines, wheat germ is rich in vitamins and minerals. The positive effect of wheat germ consumption has been reported in treatment of diseases such as cancers, obesity, diabetes, asthma, anemia, eczema, high blood pressure, ulcers, and gastritis. Wheat germ is comprised of magnesium, zinc, calcium, vitamin E, vitamin C, vitamin B_12_, vitamin B_6_, thiamin, riboflavin, niacin, folic acid, and iron (32-34). Various studies have been conducted to assess the positive effects of some wheat germ compounds (vitamins E, B_6_, and B_1_) on reducing symptoms of dysmenorrhea.

Proctor and Farquhar reported the efficiency of vitamin B_6_ in relieving the symptoms of dysmenorrhea (21). In addition, vitamin E intake reduces the intensity of dysmenorrhea (35, 36). In other studies, positive effects of vitamin B_1_ in dysmenorrhea have been addressed (37). Wheat germ has anti-inflammatory, sedative, antidepressant, and soothing properties and is rich in vitamins E, B_12_, B_6_, magnesium, zinc, and calcium.

## 2. Objectives

Considering the properties of wheat germ, this study was conducted to determine the effects of wheat germ extract on the severity and systemic manifestations of primary dysmenorrhea.

## 3. Patients and Methods

This triple-blinded randomized clinical trial was performed on 90 women working in governmental hospitals affiliated with Hamadan University of Medical Sciences (Hamadan, IR Iran) from September 2013 to March 2014.

The study protocol was approved by the Ethics Committee of Shahid Beheshti University of Medical Sciences. After getting permission from the Ethics Committee (Ethical code 116/2879; date 30/9/2013), and registering the study in the Iranian Registry of clinical trial (IRCT ID: 201310286807N8), participants were informed about the purpose and methods of the study and the interested women were invited to participate in this study. Among 624 employed women in hospitals, 312 reported primary dysmenorrhea. After more assessments, 90 individuals were enrolled in the study.

The inclusion criteria were as follows: patients with 20 to 45 years of age, body mass index (BMI) of 19.8 to 26 kg/m^2^, having no night shifts, no smoking, regular menstrual periods with 21-day to 35-day cycles and three-day to ten-day bleeding period, no identified physical or psychological diseases, no relatives death and divorce in the preceding three months, and no antidepressants, hormones, or contraceptives use in the preceding three months. The exclusion criteria were relatives’ death and divorce, adverse events, and pregnancy.

The two groups were matched for variables related to dysmenorrhea and systemic symptoms including age, weight, age at menarche, and BMI.

Sample size was estimated using the following formula:

N = (Z_α/2_ + Z_β_) ^2^σ ^2^) / ε^2^

With 95% confidence interval, a minimum sample size of 90 participants was determined.

A self-reported checklist was used to collect data on the intensity of primary dysmenorrhea, the systemic symptoms associated with dysmenorrhea, and the number of sedative drugs taken for dysmenorrhea. Its validity was confirmed through content validity, and test-retest method was used to assess its reliability (r = 0.91). First, maximum pain intensity during two consecutive menstrual cycles were recorded in forms number 2 and 3.

During the first three days of menstruation, pain intensity was measured by a visual analogue scale (VAS; a 10-cm tape) three times a day. Scores of one through three, four through seven, and eight through ten on the VAS indicated mild, moderate, and severe pain, respectively. A four-point verbal rating scale from zero to three was employed to assess the intensity of the associated systemic symptoms (fatigue, diarrhea, syncope, nausea and vomiting, lack of energy, headache, and mood swings); the validity and reliability of this scale was confirmed in various studies (37-41). After obtaining written informed consent, the table of random numbers was used to allocate the participants to any of the groups receiving wheat germ extract or placebo. 

First, the extraction was performed using ethanol 70% and repeated three times and each time for 24 hours. The dried extracts were used to produce capsules containing 400 mg of wheat germ extract. Three capsules of wheat germ extract or placebo were administered daily٫ from the 16th day through the fifth day of the next menstrual cycle for two consecutive months. The subjects received the medicine and filled out forms number 2 and 3 again.

**Figure 1.**
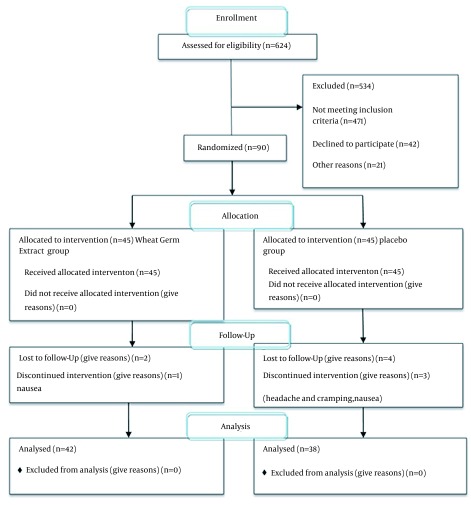
Flow of Participants Through the Research.

The subjects could use NSAIDs if required; however, they were asked to take these medications equal or longer than one hour after taking the study capsule. Patients were instructed to score and register the pain severity before consumption of the sedative. The questionnaires were collected after two months of intervention and the mean intensity of primary dysmenorrhea and its associated systemic symptoms were determined. Among 90 participants, one person was excluded due to pregnancy, three due to digestive complications, and six due to missing or inappropriately taking the medicines. The final analysis included 80 women: 42 had received wheat germ extract and 38 had received placebo (Figure 1).

Data were analyzed with SPSS v.17 (SPSS Inc., Chicago, IL, USA) using t test, GEE logistic regression, Friedman test, McNemar test with Bonferroni, and Mann-Whitney U test. The independent-samples t-test was used to compare age, weight, BMI, age at menarche, and duration of menstrual cycle between study groups. The Friedman test was employed to compare pain severity and the systemic symptoms associated with menstruation between the three periods within groups, and the Mann-Whitney U test was used to compare the findings between the study groups. If the results of the Friedman test were significant, the periods were compared in pairs via McNemar test with Bonferroni correction. P value < 0.05 was considered as statistically significant.

## 4. Results

The length of the menstrual period in all participants ranged from three to ten days and the interval between the periods ranged from 21 to 35 days. No one in the groups exercised professionally and regularly. In order to control the confounding factors, both groups were matched for age, weight, BMI, age at menarche, length of menstrual cycle, menstruation duration, and marital status and there was not a statistically significant difference between the two groups with regard to these variables (Table 1).

As Tables 2 and 3 show, the study groups were matched for the severity of the pain and systemic manifestations, and there was no statistically significant difference between them in this respect before the intervention.

The reduction in severity of pain was statistically significant only in the wheat germ extract group (P < 0.001) and there was no statistically significant change in the placebo group (Table 2). The severity of the systemic symptoms associated with dysmenorrhea had reduced in both groups; however, this reduction was significantly greater in the wheat germ extract group in comparison to placebo group (P < 0.001) (Tables 3 and 4). The results of mixed model analysis or GEE analysis are shown in Table 5. The effect of treatment group on the severity and systemic manifestations of dysmenorrhea was significant (P < 0.05).

Neither of the groups showed changes in the duration and extent of bleeding and there was no statistically significant difference between the groups in this regard. The number of consumed painkillers remarkably reduced after intervention in the wheat germ extract group (P < 0.001) while there was no statistically significant changes in the placebo group with regard to this score. In addition, 95.2% of the wheat germ extract group and 92.9% of the placebo group participants did not report any complications.

**Table 1.** Demographic Characteristics of the Study Groups ^a^

	Treatment Categories		
Variable	Wheat Germ Extract	Placebo	P Value
**Age, y**	33.452 ± 5.89	32.842 ± 5.58	0.637
**Weight, kg**	59.166 ± 9.52	58.592 ± 8.78	0.781
**BMI ** ^**b**^ **, kg/m²**	23.439 ± 1.86	23.026 ± 1.88	0.328
**Length of Menstrual Cycle, d**	27 ± 2.81	28.026 ± 2.07	0.348
**Age at Menarche, y**	13.571 ± 1.48	13.921 ± 1.44	0.289
**Marital Status**			0.625
Single	28.6	23.7	
Married	71.4	76.3	

^a^ Data are presented as mean ± SD or percent.

^b^ Body mass index.

**Table 2.** Severity of Dysmenorrhea Before and After the Intervention in Study Groups ^a, b^

Treatment Round	Before Treatment	Four Weeks After Treatment	Eight Weeks After Treatment	P Value
	Percentiles 50, (25-75)	Percentiles 50, (25-75)	Percentiles 50, (25-75)	
**Wheat Germ Extract**	4.791 (1.916-6.666)	2.121 (0.833-3.333)	0.606 (0-1.553)	0.000
**Placebo**	4.999 (1.666-6.874)	3.333 (0.75-6.666)	3.610 (0.0-0.666)	0.203
**P value**	0.509	0.04	0.003	

^a^ Man-Whitney U test.

^b^ Friedman and Bonferroni.

**Table 3.** Severity of Systemic Signs Associated With Dysmenorrhea Before and After the Intervention in Study Groups ^a,b^

Treatment Round	Before Treatment	Four Weeks After Treatment	Eight Weeks After Treatment	P Value
	Percentiles, 50 (25-75)	Percentiles, 50 (25-75)	Percentiles, 50 (25-75)	
**Wheat Germ Extract**	0.690 (0.518-1.020)	0.272 (0.168-0.428)	0.227 (0.116-0.295)	0.000
**Placebo**	0.699 (0.428-0.959)	0.541 (0.285-0.832)	0.525 (0.285-0.862)	0.20
**P value**	0.620	0.001	0.000	

^a^ Man-Whitney U test.

^b^ Friedman & Bonferroni.

**Table 4.** Severity of Systemic Signs Associated With Dysmenorrhea, as Measured on a Multidimensional Verbal Scale (Score Range 0-3) ^a^

Systemic Sign	Baseline			First Cycle			Second Cycle		
	Percentiles, 50 (25-75)			Percentiles, 50 (25-75)			Percentiles, 50 (25-75)		
	Wheat Germ	Placebo	P Value	Wheat Germ	Placebo	P Value	Wheat Germ	Placebo	P Value
**Fatigue**	1.35 (0.999-1.912)	1.292 (0.999-1.999)	0.343	0.363 (0.0-0.999)	0.999 (0.0- 1.999)	0.020	0.000 (0.0-0.818)	0.999 (0.0- 1.795)	0.001
**Nausea, Vomiting**	0.000 (0.0-0.625)	0.000 (0.0-1)	0.888	0.000 (0.0-0.082)	0.000 (0.0-1)	0.315	0.000 (0.0-0.0)	0.000 (0.0-0.330)	0.125
**Lack Of Energy**	0.000 (0.0-1)	0.000 (0.0-1)	0.979	0.000 (0.00-0.00)	0.000 (0.0-1)	0.273	0.000 (0.00-0.00)	0.000 (0.0-0.625)	0.368
**Headache**	0.0000 (0.0-1.999)	1.164 (0.0-1.999)	0.459	0.181 (0.0-0.999)	1.272 (0.0- 1.999)	0.006	0.000 (0.0-0.545)	1.136 (0.0-1.999)	0.000
**Diarrhea**	0.625 (0.0-1.342)	0.000 (0.0-1.041)	0.276	0.000 (0.0-0.999)	0.000 (0.0-0.999)	0.832	0.000 (0.0-0.0)	0.000 (0.0-0.999)	0.321
**Mood Swings**	0.999 (0.0-1.669)	0.999 (0.0-1.961)	0.767	0.545 (0.0-0.8636)	0.999 (0.0-0.999)	0.999	0.000 (0.0-0.272)	0.999 (0.0-1.525)	0.001
**Syncope**	0.000 (0.0-0.0)	0.000 (0.0-0.0)	0.223	0.000 (0.0-0.0)	0.000 (0.0-0.0)	0.569	0.000 (0.0-0.0)	0.000 (0.0-0.0)	0.291

^a^ Man-Whitney U test.

**Table 5.** GEE Model of Dysmenorrhea, Fatigue, Diarrhea, Syncope, Nausea and Vomiting, Lack of Energy, Headache, and Mood Swings

	Estimate	Standard Error	P Value
**Dysmenorrhea**			
Wheat Germ Extract	B = 1.578	0.510	0.002
Placebo		Reference group	
**Fatigue**			
Wheat Germ Extract	B = 0.328	0.1372	0.017
Placebo		Reference group	
**Nausea, Vomiting**			
Wheat Germ Extract	B = 0.129	0.1091	0.238
Placebo		Reference group	
**Lack of Energy**			
Wheat Germ Extract	B = 0.107	0.136	0.434
Placebo		Reference group	
**Headache**			
Wheat Germ Extract	B = 0.559	0.168	0.001
Placebo		Reference group	
**Diarrhea**			
Wheat Germ Extract	B = 0.055	0.1374	0.686
Placebo		Reference group	
**Mood Swings**			
Wheat Germ Extract	B = 0.276	0.1327	0.031
Placebo		Reference group	
**Syncope**			
Wheat Germ Extract	B = 0.061	0.0545	0.260
Placebo		Reference group	

## 5. Discussion

The present study was the first to investigate the use of wheat germ extract in the treatment of dysmenorrhea. The results showed that wheat germ extract reduced the severity of pain (P < 0.001). While there was no statistically significant difference in the placebo group. There was a significant difference in pain intensity between groups (P < 0.001). The severity of the systemic symptoms associated with dysmenorrhea was reduced in both groups, but this reduction was significantly greater in the wheat germ extract group in comparison with placebo group (P < 0.001). The Man-Whitney U test revealed statistically significant differences between both groups in systemic symptoms associated with dysmenorrhea including fatigue, headache, and mood swings.

Wheat germ activates neuropeptides, cytokines, and macrophages due to its anti-inflammatory properties and hence, can decrease inflammation within two weeks. Moreover, daily use of 20 to 100 mL of wheat germ extract has been suggested to alleviate the symptoms of ulcerative colitis (e.g. abdominal pain) by 78% (32). Such findings can be justified by anti-inflammatory properties of wheat which can in turn enhance blood circulation, remove blood stasis, and reduce pain (42).

Previous researches have not directly addressed the effect of wheat germ extract on dysmenorrhea. In addition, the positive effects of some components of wheat germ such as vitamin B_12_٫ thiamin, pyridoxine, magnesium, and vitamin E on the intensity of dysmenorrhea have been previously highlighted (21). Vitamin B_12_ acts as an antioxidant and lowers the levels of prostaglandins and leukotrienes; thus, it can relieve pain. The benefits of omega-3 plus vitamin B_12_ in easing menstrual pains have been thoroughly described (38). Therefore, the desirable effects of wheat germ on our participants might be due to its vitamin B_12_ content.

Wheat germ is also rich in vitamin E, an antioxidant that can mitigate the symptoms of dysmenorrhea by preventing arachidonic acid oxidation and the production of prostaglandins (36). Twice-daily supplementation with 200 units of vitamin E has been reported to reduce the duration and severity of primary dysmenorrhea (43). Therefore, the desirable effects of wheat germ on our participants might be due to its vitamin E content.

A study suggested daily intake of vitamin D (300000 IU/mL) from five days before the menstrual period to lower the pain of dysmenorrhea (44). Therefore, the desirable effects of wheat germ on our participants might be caused by vitamin D content of wheat germ. Wheat germ also contains vitamin B_1_. Receiving 100-mg vitamin B1 daily for three successive cycles has been proved to alleviate the pain as effectively as 400-mg ibuprofen without having any particular side effects (45). Magnesium is another component of wheat germ that its daily intake (4.5 mg three times a day) from seven days before through the third day after the initiation of menstrual period has been confirmed to mitigate the severity of dysmenorrhea (46). Wheat germ also contains high amounts of calcium which is beneficial for reducing cramping and pain (47).

We found wheat germ consumption soothing to headaches (a systemic symptom associated with dysmenorrhea). It seems sensible as wheat germ has high levels of magnesium and vitamin B_6_ whose effects in treating migraines and generally headaches have been proven (48). Daily use of magnesium has also been reported to have positive effects on relieving headaches (49). The use of wheat reduces pains including headache (42). Furthermore, the daily use of wheat germ has been effective in improving migraines (32, 34).

Another systemic symptom associated with dysmenorrhea is fatigue. Wheat has been suggested to boost energy (49). Daily supplementation with wheat (45 g in breakfast, 40 g between meals, and 40 g in dinner) for two months has been found to decrease the feeling of tiredness by 75% (50). The vitamin B family, magnesium, and zinc in wheat germ reduce the severity of fatigue (51). Accordingly, the vitamin B family, magnesium, and zinc content of wheat germ might be responsible for reduced fatigue levels in the current study.

Mood swings are also among the systemic symptoms accompanying dysmenorrhea. Since vitamin B_6_ acts as a cofactor during the final stages of serotonin and tryptophan synthesis (52), the sedative effects of wheat germ can be attributed to the presence of this vitamin. On the other hand, magnesium deficiency has been cited as a cause of neural system damage and many conditions including agitation, anxiety, irritability, drowsiness, weakness, insomnia, and headache (48). Hence, the positive effects of wheat germ consumption on fatigue, headaches, mood, and energy level can be caused by its magnesium content. Wheat germ has sedative properties and is effective in the treatment of diseases such as ataxia, nervous system disorders, and acute mental illnesses (32, 34). Such findings are comparable to those reported in this study.

 The number of consumed sedative tablets was remarkably reduced after intervention in the wheat germ extract group (P < 0.001) while there was no statistically significant change in the placebo group. This is an important finding because NSAIDs have numerous side effects, including nausea, vomiting, dizziness, petechiae, hyperkalemia, peripheral edema, peptic ulcers, and gastric bleeding (31).

A triple-blind clinical trial revealed that using 100 units of vitamin E every six hours for three days decreases the number of consumed sedative tablets (53), which is in accordance with our study and indicates that our results might be due to the effect of vitamin E in wheat germ.

Our study did not show a significant difference between the two groups with respect to the side effects; in fact, it is not possible to attribute any specific side effect to wheat germ in this study. The findings of this research proved the effectiveness and safety of the daily intake of wheat germ extract for reducing pain severity and systemic manifestations of dysmenorrhea. Padalia et al., in their article, entitled “Multitude potential of Wheatgrass Juice (Green Blood): An overview”, reported no side effects (54), which is in line with our findings. Based on the findings of the present study, further studies are needed to compare wheat germ extract with anti-inflammatory medications.

Herbal medicines are among the most common treatments because they are safe and reliable, and have fewer side effects than do chemical medicines. Consumption of wheat germ extract mitigated the severity of pain and systemic manifestations of dysmenorrhea. The therapeutic effects of wheat germ extract might be the consequence of the positive impact of its compounds such as vitamins B_6_ and E, magnesium, and calcium, and it seems that wheat germ extract can be recommended for treating the dysmenorrhea and its systemic manifestations.
